# Supplementary material for: Divergent abiotic spectral pathways unravel pathogen stress signals across species
Source: Nat Commun. 2021 Oct 19;12:6088. doi: 10.1038/s41467-021-26335-3 (PMC8526582; doi:10.1038/s41467-021-26335-3)
Supplement: Supplementary file 1 — Supplementary Information [file 41467_2021_26335_MOESM1_ESM.pdf]

Supplementary Material

Supplementary Table 1. Narrow-band hyperspectral indices derived from hyperspectral and thermal data included in this study and their formulations.

Hyperspectral indices	Equation	Reference
**Structural indices**		
Normalised Difference Veg. Index	$NDVI = (R_{800} - R_{670})/(R_{800} + R_{670})$	Rouse *et al.* (1974)[1]
Renormalised Difference Veg. Index	$RDVI = (R_{800} - R_{670})/\sqrt{(R_{800} + R_{670})}$	Roujean & Breon (1995)[2]
Optimised Soil-Adjusted Veg. Index	$OSAVI = ((1 + 0.16) \cdot (R_{800} - R_{670})/(R_{800} + R_{670} + 0.16))$	Rondeaux *et al.* (1996)[3]
Modified Soil-Adjusted Vegetation Index	$MSAVI = \frac{2 \cdot R_{800} + 1 - \sqrt{(2 \cdot R_{800} + 1)^2 - 8(R_{800} - R_{670})}}{2}$	Qi *et al.* (1994)[4]
Triangular Vegetation Index	$TVI = 0.5 \cdot [120 \cdot (R_{750} - R_{550}) - 200 \cdot (R_{670} - R_{550})]$	Broge & Leblanc (2001)[5]
Modified Triangular Veg. Index 1	$MTVI1 = 1.2[1.2(R_{800} - R_{550}) - 2.5(R_{670} - R_{550})]$	Haboudane *et al.* (2004)[6]
Modified Triangular Veg. Index 2	$MTVI2 = \frac{1.5[1.2(R_{800} - R_{550}) - 2.5(R_{670} - R_{550})]}{\sqrt{(2R_{800} + 1)^2 - (6R_{800} - 5\sqrt{R_{670}}) - 0.5}}$	Haboudane *et al.* (2004)[6]
Modified Chlorophyll Abs. Index	$MCARI = [(R_{700} - R_{670}) - 0.2(R_{700} - R_{550})] \cdot (R_{700}/R_{670})$	Haboudane *et al.* (2004)[6]
Modified Chlorophyll Abs. Index 1	$MCARI1 = 1.2[2.5(R_{800} - R_{670}) - 1.3(R_{800} - R_{550})]$	Haboudane *et al.* (2004)[6]
Modified Chlorophyll Abs. Index 2	$MCARI2 = \frac{1.5[2.5(R_{800} - R_{670}) - 1.3(R_{800} - R_{550})]}{\sqrt{(2R_{800} + 1)^2 - (6R_{800} - 5\sqrt{R_{670}}) - 0.5}}$	Haboudane *et al.* (2004)[6]
Simple Ratio	$SR = R_{800}/R_{670}$	Jordan (1969)[7]
Modified Simple Ratio	$MSR = \frac{R_{800}/R_{670} - 1}{(R_{800}/R_{670})^{0.5} + 1}$	Chen (1996)[8]
Enhanced Vegetation Index	$EVI = 2.5 \cdot (R_{800} - R_{670})/(R_{800} + 6 \cdot R_{670} - 7.5 \cdot R_{800} + 1)$	Liu & Huete (1995)[9]
**Pigment indices**		
Vogelmann indices	$VOG1 = R_{740}/R_{720}$	Vogelmann *et al.* (1993)[10]
	$VOG2 = (R_{734} - R_{747})/(R_{715} + R_{726})$	Vogelmann *et al.* (1993)[10]
	$VOG3 = (R_{734} - R_{747})/(R_{715} + R_{720})$	Vogelmann *et al.* (1993)[10]
Gitelson & Merzlyak indices	$GM1 = R_{750}/R_{550}$	Gitelson & Merzlyak (1997)[11]
	$GM2 = R_{750}/R_{700}$	Gitelson & Merzlyak (1997)[11]
Transformed Chlorophyll Absorption in Reflectance Index	$TCARI = 3 \cdot [(R_{700} - R_{670}) - 0.2 \cdot (R_{700} - R_{550}) \cdot (R_{700}/R_{670})$	Haboudane *et al.* (2002)[12]
Transformed Chlorophyll Absorption in Reflectance Index/ Optimised Soil-Adjusted Vegetation Index	$\frac{TCARI}{OSAVI} = \frac{3 \cdot [(R_{700} - R_{670}) - 0.2 \cdot (R_{700} - R_{550}) \cdot (R_{700}/R_{670})}{((1 + 0.16) \cdot (R_{800} - R_{670})/(R_{800} + R_{670} + 0.16))}$	Haboudane *et al.* (2002)[12]
Chlorophyll Index Red Edge	$CI = R_{750}/R_{710}$	Haboudane *et al.* (2002)[12]
Simple Ratio Pigment Index	$SRPI = R_{430}/R_{680}$	Peñuelas *et al.* (1995)[13] Barnes *et al.* (1992)[14]
Normalised Phaeophytinization Index	$NPQI = (R_{415} - R_{435})/(R_{415} + R_{435})$	Peñuelas *et al.* (1995)[13] Barnes *et al.*, 1992)[14]
Normalised Pigments Index	$NPCI = (R_{680} - R_{430})/(R_{680} + R_{430})$	Peñuelas *et al.* (1995)[13]
Carter indices	$CTRI1 = R_{695}/R_{420}$	Carter (1994)[15]
	$CAR = R_{695}/R_{760}$	Carter *et al.* (1996)[16]
Reflectance band ratio indices	$DCabCxc = R_{672}/(R_{550} \cdot 3R_{708})$	Datt *et al.* (1998)[17]
	$DNIRCabCxc = R_{860}/(R_{550} \cdot R_{708})$	Datt *et al.* (1998)[17]
Structure-Intensive Pigment Index	$SIPI = (R_{800} - R_{445})/(R_{800} + R_{680})$	Peñuelas *et al.* (1995)[13]
Carotenoid Reflectance Indices	$CRI_{550} = (1/R_{510}) - (1/R_{550})$	Gitelson *et al.* (2003; 2006)[18,19]
	$CRI_{700} = (1/R_{510}) - (1/R_{700})$	Gitelson *et al.* (2003; 2006)[18,19]
	$CRI_{550\_515} = (1/R_{515}) - (1/R_{550})$	Gitelson *et al.* (2006)[19]
	$CRI_{700\_515} = (1/R_{515}) - (1/R_{700})$	Gitelson *et al.* (2006)[19]
	$RNIR \cdot CRI_{550} = (1/R_{510}) - (1/R_{550}) \cdot R_{770}$	Gitelson *et al.* (2003; 2006)[18,19]
	$RNIR \cdot CRI_{700} = (1/R_{510}) - (1/R_{700}) \cdot R_{770}$	Gitelson *et al.* (2003; 2006)[18,19]
Plant Senescencing Reflectance Index	$PSRI = (R_{680} - R_{500})/R_{750}$	Merzlyak *et al.* (1999)[20]

Pigment Specific Simple Ratio Chlorophyll a	$PSSRa = R_{800}/R_{675}$	Blackburn (1998)[21]
Pigment Spec. Simple Ratio Chl. b	$PSSRb = R_{800}/R_{650}$	Blackburn (1998)[21]
Pigment Specific Simple Ratio Carot.	$PSRRc = R_{800}/R_{500}$	Blackburn (1998)[21]
Pigment Specific Normalised Difference	$PSNDc = (R_{800} - R_{470})/(R_{800} + R_{470})$	Blackburn (1998)[21]
**Xanthophyll indices**		
Photochemical Refl. Index (570)	$PRI_{570} = (R_{570} - R_{531})/(R_{570} + R_{531})$	Gamon *et al*. (1992)[22]
Photochemical Refl. Index (515)	$PRI_{515} = (R_{515} - R_{531})/(R_{515} + R_{531})$	Hernández-Clemente *et al*. (2011)[23]
Photochemical Refl. Index (512)	$PRI_{m1} = (R_{512} - R_{531})/(R_{512} + R_{531})$	Hernández-Clemente *et al*. (2011)[23]
Photochemical Refl. Index (600)	$PRI_{m2} = (R_{600} - R_{531})/(R_{600} + R_{531})$	Gamon *et al*. (1992)[22]
Photochemical Refl. Index (670)	$PRI_{m3} = (R_{670} - R_{531})/(R_{670} + R_{531})$	Gamon *et al*. (1992)[22]
Photochemical Refl. Index (670 and 570)	$PRI_{m4} = (R_{570} - R_{531} - R_{670})/(R_{570} + R_{531} + R_{670})$	Hernández-Clemente *et al*. (2011)[23]
Normalised Photoch. Refl. Index	$PRI_n = PRI_{570}/[RDVI \cdot (R_{700}/R_{670})$	Zarco-Tejada *et al*. (2013)[24]
Carotenoid/Chlorophyll Ratio Index	$PRI \cdot CI = (R_{570} - R_{530})/(R_{570} + R_{530}) \cdot ((R_{760}/R_{700}) - 1)$	Garrity *et al*. (2011)[25]
**R/G/B indices**		
Redness Index	$R = R_{700}/R_{670}$	Gitelson *et al*. (2000)[26]
Greenness Index	$G = R_{570}/R_{670}$	Calderon *et al*. (2013)[27]
Blue Index	$B = R_{450}/R_{490}$	Calderon *et al*. (2013)[27]
Blue/green indices	$BGI1 = R_{400}/R_{550}$	Zarco-Tejada *et al*. (2005)[28]
	$BGI2 = R_{450}/R_{550}$	Zarco-Tejada *et al*. (2005)[28]
Blue/red indices	$BRI1 = R_{400}/R_{690}$	Zarco-Tejada *et al*. (2012)[29]
	$BRI2 = R_{450}/R_{690}$	Zarco-Tejada *et al*. (2012)[29]
BF1	$BF1 = R_{400}/R_{410}$	Zarco-Tejada *et al*. (2018)[30]
BF2	$BF2 = R_{400}/R_{420}$	Zarco-Tejada *et al*. (2018)[30]
BF3	$BF3 = R_{400}/R_{430}$	Zarco-Tejada *et al*. (2018)[30]
BF4	$BF4 = R_{400}/R_{440}$	Zarco-Tejada *et al*. (2018)[30]
BF5	$BF5 = R_{400}/R_{450}$	Zarco-Tejada *et al*. (2018)[30]
Red/green indices	$RGI = R_{690}/R_{550}$	Zarco-Tejada *et al*. (2005)[28]
Ratio Analysis of Reflectance Spectra	$RARS = R_{746}/R_{513}$	Chappelle *et al*. (1992)[31]
Lichtenthaler Index	$LIC1 = (R_{800} - R_{680})/(R_{800} + R_{680})]$	Lichtenhaler *et al*. (1996)[32]
	$LIC2 = R_{440}/R_{690}$	Lichtenhaler *et al*. (1996)[32]
	$LIC3 = R_{440}/R_{740}$	Lichtenhaler *et al*. (1996)[32]
**Chlorophyll fluorescence**		
Reflectance Curvature Index	$CUR = (R_{675} \cdot R_{690})/R_{683}^2$	Zarco-Tejada *et al*. (2000)[33]
Fraunhofer Line Depth (FLD) principle	$FLD = \frac{E_{out} \cdot L_{in} - E_{in} \cdot L_{out}}{E_{out} - E_{in}}$	Plascyk (1975)[34] see Mohammed *et al.* (2019)[35]
**Plant disease index**		
Healthy-index	$HI = \frac{(R_{534} - R_{698})/}{R_{534} + R_{698}} - \frac{1}{2} \cdot R_{704}$	Mahlein *et al*. (2012)[36]
**Thermal index**		
Crop Water Stress Index (CWSI)	$CWSI = \frac{(T_c - T_a) - (T_c - T_a)_{LL}}{(T_c - T_a)_{UL} - (T_c - T_a)_{LL})}$ LL, UL = lower and upper limits, respectively	Idso *et al.* (1981)[37]

Supplementary Table 2. Primer sequences for the PCR and qPCR techniques used for *Verticillium dahliae* (*Vd*) and *Xylella fastidiosa* (*Xf*) in this study.

Protocol	Target Pathogen	Primers	Sequence
PCR	*V. dahliae*	Forward primer DB19	5'-CGGTGACATAATACTGAGAG-3′
		Reverse primer DB22	5'-GACGATGCGGATTGAACGAA-3′
		Internal primer	5'-TGAGACTCGGCTGCCACAC-3′
qPCR	*X. fastidiosa*	Forward primer XF-F	5′-CACGGCTGGTAACGGAAGA-3′
		Reverse primer XF-R	5′-GGGTTGCGTGGTGAAATCAAG-3′
		Probe XF-P	5′-6-FAM-TCGCATCCCGTGGCTCAGTCC-BHQ-1-3′

Supplementary Table 3. Values and ranges used for the model inversion and look-up-table (LUT) generation for the PROSAIL (PROSPECT-D + 4SAIL) radiative transfer model.

Parameter	Abbreviation	Value / range
Chlorophyll content [μg/cm²]	C_{a+b}	10–70
Carotenoid content [μg/cm²]	C_{x+c}	0–20
Anthocyanin content [μg/cm²]	Anth	0–7.5
Dry matter content [g/cm²]	C_m	0.012
Water content [g/cm²]	C_w	0.009
Mesophyll struct. coeff.	N	1–2.5
Leaf Area Index [m²/m²]	LAI	0.3–5
Average leaf angle [deg.]	$Lidf_a$	0–90
Hot spot parameter	Hot	0.01
Soil reflectance	R_{soil}	-
Observer angle [deg.]	tto	0
Sun zenith angle [deg.]	tts	0–53.75
Relative azimuth angle [deg.]	psi	0

Supplementary References

1. Rouse, J. W., Jr., Haas, R. H., Schell, J. A. & Deering, D. W. Monitoring Vegetation Systems in the Great Plains with Erts. *NASA Special Publication* **351**, 309 (1974).
2. Roujean, J.-L. & Breon, F.-M. Estimating PAR absorbed by vegetation from bidirectional reflectance measurements. *Remote Sensing of Environment* **51**, 375–384 (1995).
3. Rondeaux, G., Steven, M. & Baret, F. Optimization of soil-adjusted vegetation indices. *Remote Sensing of Environment* **55**, 95–107 (1996).
4. Qi, J., Chehbouni, A., Huete, A. R., Kerr, Y. H. & Sorooshian, S. A modified soil adjusted vegetation index. *Remote Sensing of Environment* **48**, 119–126 (1994).

5. Broge, N. H. & Leblanc, E. Comparing prediction power and stability of broadband and hyperspectral vegetation indices for estimation of green leaf area index and canopy chlorophyll density. *Remote Sensing of Environment* **76**, 156–172 (2001).
6. Haboudane, D., Miller, J. R., Pattey, E., Zarco-Tejada, P. J. & Strachan, I. B. Hyperspectral vegetation indices and novel algorithms for predicting green LAI of crop canopies: Modeling and validation in the context of precision agriculture. *Remote Sensing of Environment* **90**, 337–352 (2004).
7. Jordan, C. F. Derivation of Leaf-Area Index from Quality of Light on the Forest Floor. *Ecology* **50**, 663–666 (1969).
8. Chen, J. M. Evaluation of Vegetation Indices and a Modified Simple Ratio for Boreal Applications. *Canadian Journal of Remote Sensing* **22**, 229–242 (1996).
9. Liu, H. Q. & Huete, A. Feedback based modification of the NDVI to minimize canopy background and atmospheric noise. *IEEE Transactions on Geoscience and Remote Sensing* **33**, 457–465 (1995).
10. Vogelmann, T. C. Plant Tissue Optics. *Annu. Rev. Plant. Physiol. Plant. Mol. Biol.* **44**, 231–251 (1993).
11. Gitelson, A. A. & Merzlyak, M. N. Signature Analysis of Leaf Reflectance Spectra: Algorithm Development for Remote Sensing of Chlorophyll. *Journal of Plant Physiology* **148**, 494–500 (1996).
12. Haboudane, D., Miller, J. R., Tremblay, N., Zarco-Tejada, P. J. & Dextraze, L. Integrated narrow-band vegetation indices for prediction of crop chlorophyll content for application to precision agriculture. *Remote Sensing of Environment* **81**, 416–426 (2002).
13. Penuelas, J., Frederic, B. & Filella, I. *Semi-Empirical Indices to Assess Carotenoids/Chlorophyll-a Ratio from Leaf Spectral Reflectance*. vol. 31 (1995).
14. Barnes, J. D., Balaguer, L., Manrique, E., Elvira, S. & Davison, A. W. A reappraisal of the use of DMSO for the extraction and determination of chlorophylls a and b in lichens and higher plants. *Environmental and Experimental Botany* **32**, 85–100 (1992).
15. Carter, G. A. Ratios of leaf reflectances in narrow wavebands as indicators of plant stress. *International Journal of Remote Sensing* **15**, 697–703 (1994).
16. Carter, G. A., Dell, T. R. & Cibula, W. G. Spectral reflectance characteristics and digital imagery of a pine needle blight in the southeastern United States. *Canadian journal of forest research* (1996).
17. Datt, B. Remote Sensing of Chlorophyll a, Chlorophyll b, Chlorophyll a+b, and Total Carotenoid Content in Eucalyptus Leaves. *Remote Sensing of Environment* **66**, 111–121 (1998).
18. Gitelson, A. A., Gritz †, Y. & Merzlyak, M. N. Relationships between leaf chlorophyll content and spectral reflectance and algorithms for non-destructive chlorophyll assessment in higher plant leaves. *Journal of Plant Physiology* **160**, 271–282 (2003).
19. Gitelson, A. A., Keydan, G. P. & Merzlyak, M. N. Three-band model for noninvasive estimation of chlorophyll, carotenoids, and anthocyanin contents in higher plant leaves. *Geophys. Res. Lett.* **33**, L11402 (2006).
20. Merzlyak, M. N., Gitelson, A. A., Chivkunova, O. B. & Rakitin, V. YU. Non-destructive optical detection of pigment changes during leaf senescence and fruit ripening. *Physiologia Plantarum* **106**, 135–141 (1999).
21. Blackburn, G. A. Spectral indices for estimating photosynthetic pigment concentrations: A test using senescent tree leaves. *International Journal of Remote Sensing* **19**, 657–675 (1998).

22. Gamon, J. A., Peñuelas, J. & Field, C. B. A narrow-waveband spectral index that tracks diurnal changes in photosynthetic efficiency. *Remote Sensing of Environment* **41**, 35–44 (1992).
23. Hernández-Clemente, R., Navarro-Cerrillo, R. M., Suárez, L., Morales, F. & Zarco-Tejada, P. J. Assessing structural effects on PRI for stress detection in conifer forests. *Remote Sensing of Environment* **115**, 2360–2375 (2011).
24. Zarco-Tejada, P. J., Morales, A., Testi, L. & Villalobos, F. J. Spatio-temporal patterns of chlorophyll fluorescence and physiological and structural indices acquired from hyperspectral imagery as compared with carbon fluxes measured with eddy covariance. *Remote Sensing of Environment* **133**, 102–115 (2013).
25. Garrity, S. R., Eitel, J. U. H. & Vierling, L. A. Disentangling the relationships between plant pigments and the photochemical reflectance index reveals a new approach for remote estimation of carotenoid content. *Remote Sensing of Environment* **115**, 628–635 (2011).
26. Gitelson, A. A. *et al.* Remote Estimation of Phytoplankton Density in Productive Waters. *Arch. HydrobioI. Spec. Issues Advanc. Limnol. Limnology and Lake Management 2000+* **55**, 121–136 (2000).
27. Calderón, R., Navas-Cortés, J. A., Lucena, C. & Zarco-Tejada, P. J. High-resolution airborne hyperspectral and thermal imagery for early detection of Verticillium wilt of olive using fluorescence, temperature and narrow-band spectral indices. *Remote Sensing of Environment* **139**, 231–245 (2013).
28. Zarco-Tejada, P. J. *et al.* Assessing vineyard condition with hyperspectral indices: Leaf and canopy reflectance simulation in a row-structured discontinuous canopy. *Remote Sensing of Environment* **99**, 271–287 (2005).
29. Zarco-Tejada, P. J., González-Dugo, V. & Berni, J. A. J. Fluorescence, temperature and narrow-band indices acquired from a UAV platform for water stress detection using a micro-hyperspectral imager and a thermal camera. *Remote Sensing of Environment* **117**, 322–337 (2012).
30. Zarco-Tejada, P. J. *et al.* Previsual symptoms of Xylella fastidiosa infection revealed in spectral plant-trait alterations. *Nature Plants* (2018) doi:10.1038/s41477-018-0189-7.
31. Chappelle, E. W., Kim, M. S. & McMurtrey, J. E. Ratio analysis of reflectance spectra (RARS): An algorithm for the remote estimation of the concentrations of chlorophyll A, chlorophyll B, and carotenoids in soybean leaves. *Remote Sensing of Environment* **39**, 239–247 (1992).
32. Lichtenthaler, H. K. Vegetation Stress: an Introduction to the Stress Concept in Plants. *Journal of Plant Physiology* **148**, 4–14 (1996).
33. Zarco-Tejada, P. J., Miller, J. R., Mohammed, G. H. & Noland, T. L. Chlorophyll Fluorescence Effects on Vegetation Apparent Reflectance: I. Leaf-Level Measurements and Model Simulation. *Remote Sensing of Environment* **74**, 582–595 (2000).
34. Plascyk, J. A. The MK II Fraunhofer Line Discriminator (FLD-II) for Airborne and Orbital Remote Sensing of Solar-Stimulated Luminescence. *OE, OPEGAR* **14**, 144339 (1975).
35. Mohammed, G. H. *et al.* Remote sensing of solar-induced chlorophyll fluorescence (SIF) in vegetation: 50 years of progress. *Remote Sensing of Environment* **231**, 111177 (2019).
36. Mahlein, A.-K., Oerke, E.-C., Steiner, U. & Dehne, H.-W. Recent advances in sensing plant diseases for precision crop protection. *Eur J Plant Pathol* **133**, 197–209 (2012).
37. Idso, S. B., Jackson, R. D., Pinter, P. J., Reginato, R. J. & Hatfield, J. L. Normalizing the stress-degree-day parameter for environmental variability. *Agricultural Meteorology* **24**, 45–55 (1981).
