# Supplementary material for: Divergent abiotic spectral pathways unravel pathogen stress signals across species
Source: Nat Commun. 2021 Oct 19;12:6088. doi: 10.1038/s41467-021-26335-3 (PMC8526582; doi:10.1038/s41467-021-26335-3)
Supplement: Supplementary file 3 — Reporting Summary [file 41467_2021_26335_MOESM3_ESM.pdf]

nature portfolio

Corresponding author(s): Pablo J. Zarco-Tejada

Last updated by author(s): Sep 29, 2021

Reporting Summary

Nature Portfolio wishes to improve the reproducibility of the work that we publish. This form provides structure for consistency and transparency in reporting. For further information on Nature Portfolio policies, see our Editorial Policies and the Editorial Policy Checklist.

Statistics

For all statistical analyses, confirm that the following items are present in the figure legend, table legend, main text, or Methods section.

n/a	Confirmed	
☐	☒	The exact sample size (*n*) for each experimental group/condition, given as a discrete number and unit of measurement
☐	☒	A statement on whether measurements were taken from distinct samples or whether the same sample was measured repeatedly
☐	☒	The statistical test(s) used AND whether they are one- or two-sided<br>*Only common tests should be described solely by name; describe more complex techniques in the Methods section.*
☐	☒	A description of all covariates tested
☒	☐	A description of any assumptions or corrections, such as tests of normality and adjustment for multiple comparisons
☐	☒	A full description of the statistical parameters including central tendency (e.g. means) or other basic estimates (e.g. regression coefficient) AND variation (e.g. standard deviation) or associated estimates of uncertainty (e.g. confidence intervals)
☐	☒	For null hypothesis testing, the test statistic (e.g. *F*, *t*, *r*) with confidence intervals, effect sizes, degrees of freedom and *P* value noted<br>*Give P values as exact values whenever suitable.*
☒	☐	For Bayesian analysis, information on the choice of priors and Markov chain Monte Carlo settings
☒	☐	For hierarchical and complex designs, identification of the appropriate level for tests and full reporting of outcomes
☒	☐	Estimates of effect sizes (e.g. Cohen's *d*, Pearson's *r*), indicating how they were calculated

Our web collection on statistics for biologists contains articles on many of the points above.

Software and code

Policy information about availability of computer code

Data collection	Commercial software used for data collection as part of the remote sensing suite of instruments and hyperspectral cameras used in this study.
Data analysis	Matlab software (Mathworks Inc., Matick, MA, USA), version R2019b, including the following toolbox: Statistics and Machine Learning toolbox and Deep Learning toolbox.<br>R Software (R Development Core Team,Vienna, Austria) version 3.5.3, including the following packages: kernlab, Caret and viRandomForest.<br>PROSPECT-D, 4SAIL and PROSAIL radiative transfer models were implemented to retrieve plant traits using hyperspectral data. These models and the source code are freely available from the authors and accessible from repository sites online.

For manuscripts utilizing custom algorithms or software that are central to the research but not yet described in published literature, software must be made available to editors and reviewers. We strongly encourage code deposition in a community repository (e.g. GitHub). See the Nature Portfolio guidelines for submitting code & software for further information.

Data

Policy information about availability of data

All manuscripts must include a data availability statement. This statement should provide the following information, where applicable:

- Accession codes, unique identifiers, or web links for publicly available datasets
- A description of any restrictions on data availability
- For clinical datasets or third party data, please ensure that the statement adheres to our policy

A full code and data availability statement has been included. Data, computer codes and algorithms will be made available before publication at the GitHub and Zenodo repositories, including the DOI identifier.

Data Availability. The data used in this study74 are available at the repository https://github.com/HyperSens/HyperSens-Divergent-spectral-responses-Nature-Communications and can be cited as https://doi.org/10.5281/zenodo.5535095
Due to the large size of the airborne hyperspectral datasets used in this study, the image shown in Figure 1 is available upon reasonable request contacting P.J.Z.-T.

Code Availability. The codes used for this study74 are available at the repository https://github.com/HyperSens/HyperSens-Divergent-spectral-responses-Nature-Communications and can be cited as https://doi.org/10.5281/zenodo.5535095
Additional algorithms required for specific intermediate steps are available upon reasonable request contacting P.J.Z.-T.

Field-specific reporting

Please select the one below that is the best fit for your research. If you are not sure, read the appropriate sections before making your selection.

☒ Life sciences ☐ Behavioural & social sciences ☐ Ecological, evolutionary & environmental sciences

For a reference copy of the document with all sections, see nature.com/documents/nr-reporting-summary-flat.pdf

Life sciences study design

All studies must disclose on these points even when the disclosure is negative.

Sample size

Xf and Vd biotic stress datasets:
Visual assessment of the disease severity (DS) for both Xylella fastidiosa (Xf) and Verticillium dahliae (Vd) infections was performed on a 0-4 rating scale. Visual assessment of Xf-infections was performed over 7296 olive trees located in Puglia, where 4045 were evaluated as asymptomatic (DS=0) and 3251 as symptomatic (DS≥1). In addition to the visual assessments, quantitative PCR (qPCR) analyses were performed, where a total of 77 olive trees were tested.
Visual assessment of Xf-infections was performed over 943 and 4048 almond trees located in Alicante and Majorca, respectively. Furthermore, qPCR tests and AmplifyRP XRT+ assays were carried out over 226 and 265 almond trees in Alicante and Majorca, respectively. In Alicante, 430 trees were identified as asymptomatic (DS=0) and 593 as symptomatic (DS≥1). In Majorca, 2661 trees were reported as asymptomatic (DS=0) and 1387 trees were rated as symptomatic (DS≥1).
On the other hand, visual assessment of Vd-infections was performed over olive trees in two locations of Spain, i.e. Castro del Rio and Ecija. In Castro del Rio, 1878 olive trees were assessed where 1569 trees were evaluated as asymptomatic (DS=0) and 283 as symptomatic (DS≥1). In Ecija, 5223 olive trees were evaluated, where 5040 trees were identified as asymptomatic and 183 as symptomatic (DS≥1).
Field plots were selected after inspecting a wide range of fields affected by both diseases within the time of the airborne flights over the infected areas of study. This resulted in the sample size indicated above.

Abiotic stress datasets:
Olive and almond groves located outside infected areas were evaluated to assess the alterations due to abiotic-induced stress. A total of 5840 olive and 4027 olive trees were studied over a two-year period using hyperspectral and thermal images. An additional study site for validation was assessed consisting of 21,071 olive trees. This resulted in the sample size indicated above.

Data exclusions

No data reduction or data excusion was carried out

Replication

Replication of the methods and validity of the models was carried out in all data collection experiment. Models and spectral plant-trait indicators were validated over qPCR analysis, which were not included in the training dataset.

Randomization

Trees form each study site were used to evaluate biotic and abiotic induced stress.

Blinding

Researchers in the field evaluated the trees without prior knowledge of disease status, as visual symptoms were evaluated for each and every single tree. The final validation of the results was performed over qPCR test carried out later on the laboratory.

Reporting for specific materials, systems and methods

We require information from authors about some types of materials, experimental systems and methods used in many studies. Here, indicate whether each material, system or method listed is relevant to your study. If you are not sure if a list item applies to your research, read the appropriate section before selecting a response.

Materials & experimental systems

n/a	Involved in the study
☒	☐ Antibodies
☒	☐ Eukaryotic cell lines
☒	☐ Palaeontology and archaeology
☒	☐ Animals and other organisms
☒	☐ Human research participants
☒	☐ Clinical data
☒	☐ Dual use research of concern

Methods

n/a	Involved in the study
☒	☐ ChIP-seq
☒	☐ Flow cytometry
☒	☐ MRI-based neuroimaging
